# Massive Cecal Variceal Hemorrhage Treated with Transjugular Intrahepatic Portosystemic Shunt with Right Colic Vein and Ileocolic Vein Embolization

**DOI:** 10.7759/cureus.4392

**Published:** 2019-04-05

**Authors:** Tej I Mehta, Dillon Clarey, Joshua Plorde, Jay Patel, Douglas Yim

**Affiliations:** 1 Radiology, University of South Dakota Sanford School of Medicine, Sioux Falls, USA; 2 Dermatology, University of South Dakota Sanford School of Medicine, Sioux Falls, USA; 3 Interventional Radiology, Avera Medical Group, Sioux Falls, USA; 4 Interventional Radiology, Avera McKennan Hospital and University Health Centre, Sioux Falls, USA; 5 Interventional Radiology, Avera McKennan Hospital and University Health Center, Sioux Falls, USA

**Keywords:** variceal bleed, varices, transjugular intrahepatic portosystemic shunt, embolization, cecum, portal hypertension, liver, colon

## Abstract

A 40-year-old male suffering from hallucinations and bizarre behavior was brought to our emergency room (ER) by the police. His drug and alcohol screens were positive for amphetamines and a blood alcohol content of 0.029 mg/dL. His past medical history was significant for alcohol use disorder, end-stage liver disease, ascites, esophageal varices, portal hypertension, and hepatic encephalopathy. He was admitted in an encephalopathic state and developed worsening hematochezia and hemodynamic instability over the course of days. Multiple investigations including contrast enhanced computed tomography (CT), upper and lower endoscopy, and mesenteric angiography did not identify a clear cause of the bleeding. Eventually, his source of bleeding was found to be from cecal varices. A transjugular intrahepatic portosystemic shunt procedure and coil embolization of the right colic and ileocolic veins stabilized the patient and he was discharged home a few days later.

## Introduction

Cecal varices are a poorly characterized cause of gastrointestinal bleeding and are rare compared to varices in other locations in the gastrointestinal tract [1]. They usually arise secondary to alcohol-related cirrhosis and may quickly transition from benign to implacable [2]. This case demonstrates a presentation, diagnosis, and treatment of cecal varices and serves to highlight the diagnostic and treatment difficulties for this entity.

## Case presentation

A 40-year-old male suffering from hallucinations and bizarre behavior was brought by police to our emergency room (ER). His vitals on arrival were: temperature 36.9°C, pulse 124 BPM, respiration 20 per minute, blood pressure 104/57, and pulse oximetry 95% on room air. A urine drug screen was positive for amphetamines and his blood alcohol level was 0.029 mg/dL. His past medical history was significant for alcohol use disorder, end-stage liver disease, portal hypertension, ascites, esophageal varices, and hepatic encephalopathy. On examination, the patient was lethargic and difficult to arouse with an ammonia level of 109.5 umol/L. He was admitted for acute treatment of hepatic encephalopathy but developed hematochezia within 24 h of admission. An esophagogastroduodenoscopy (EGD) demonstrated grade II esophageal varices, which were banded, and portal hypertensive gastropathy. This seemed to resolve the hematochezia; however, two days later he had another episode of bright red blood per rectum. Sigmoidoscopy was performed, which demonstrated nonbleeding internal hemorrhoids. Over the next 36 h the patient complained of increasing lower abdominal pain and had intermittently bloody stools; however, a computed tomography (CT) scan of the abdomen and pelvis was negative for any acute changes. He then had two large, bloody stools and developed hypotension overnight; additionally his creatinine increased from 0.6 to 1.2 within 12 h. Given the intermittent nature of his gastrointestinal bleeding, a Model for End-Stage Liver Disease (MELD) score of 20 and concerns that he may have been developing hepatorenal syndrome, the gastroenterologist determined colonoscopy too risky. Instead, a tagged red blood cell scan was ordered as a less invasive modality to seek out intermittent bleeding. It showed abnormalities in the duodenum and stomach as well as bleeding from the right colon. The patient was taken to interventional radiology for a mesenteric angiogram. No active bleeding was identified; however, the portal venous phase of the superior mesenteric arteriogram did show dilated varices within the mesentery of the right colon.

**Figure 1:**
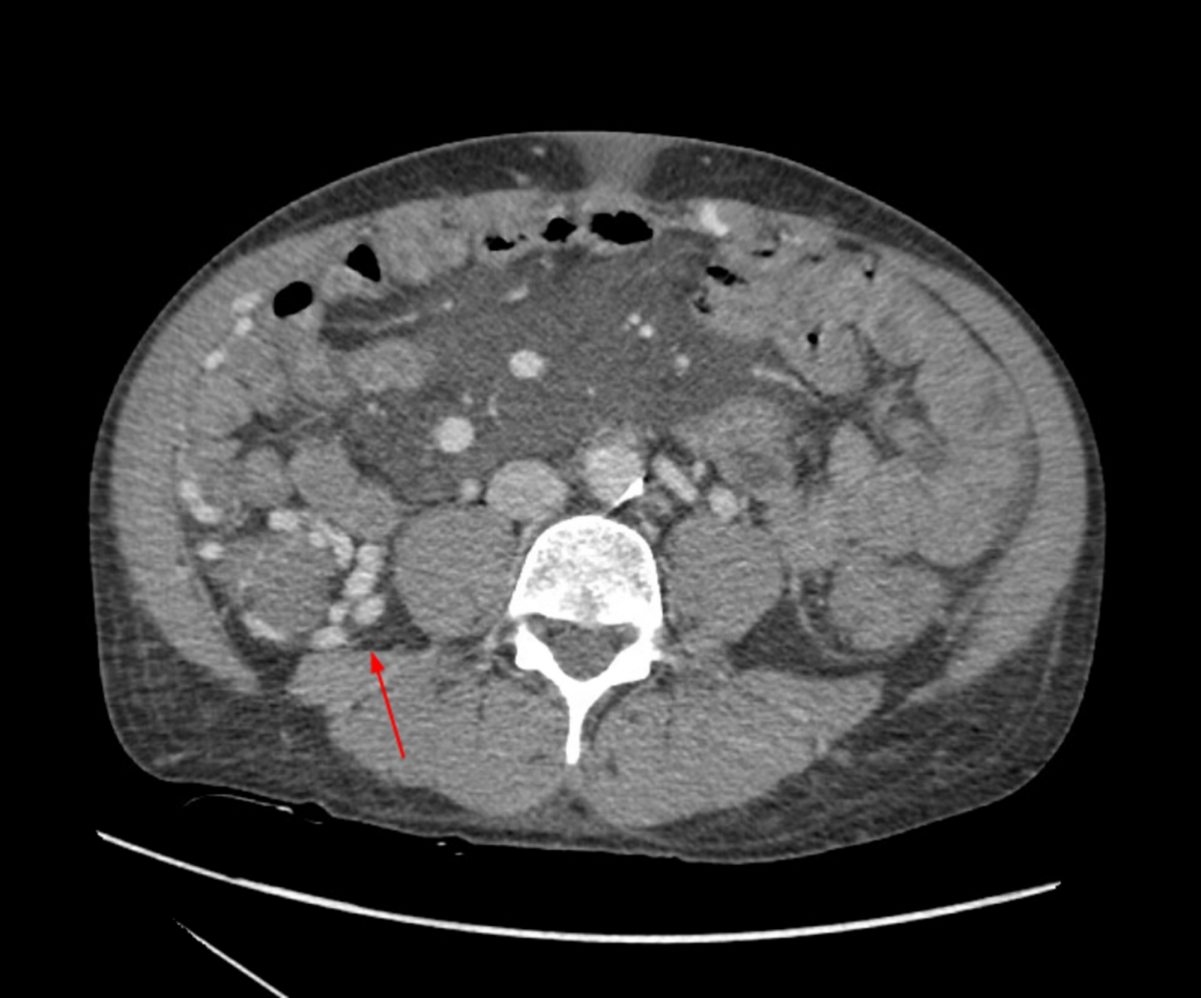
Arterial phase CT angiography demonstrating right cecal varices (red arrow) without evidence of active hemorrhage. Contrast evident within venous system during arterial phase.

**Figure 2:**
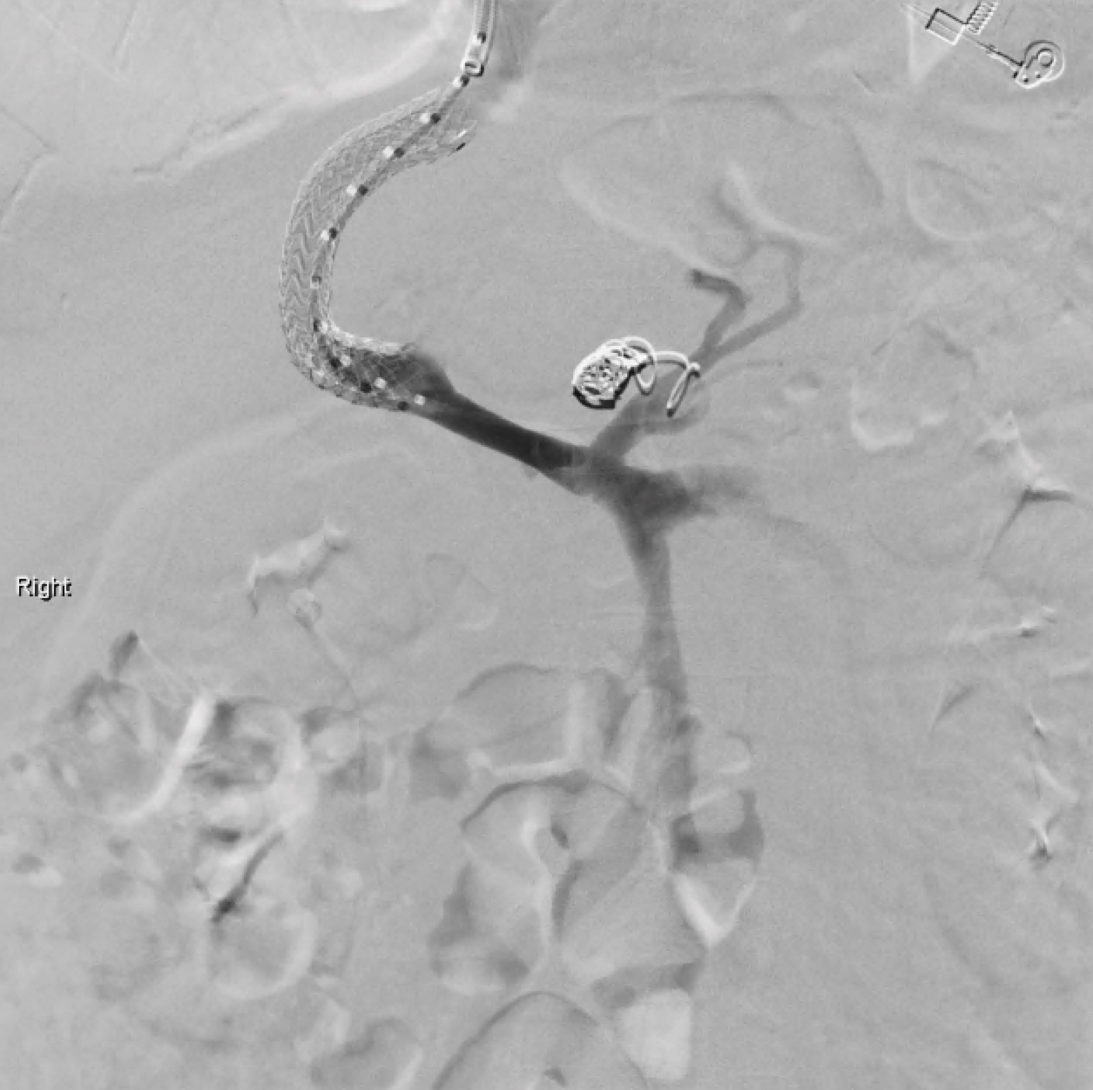
Transjugular intrahepatic portosystemic shunt (TIPS) placement. Coronary vein embolized with multiple coils.

**Figure 3:**
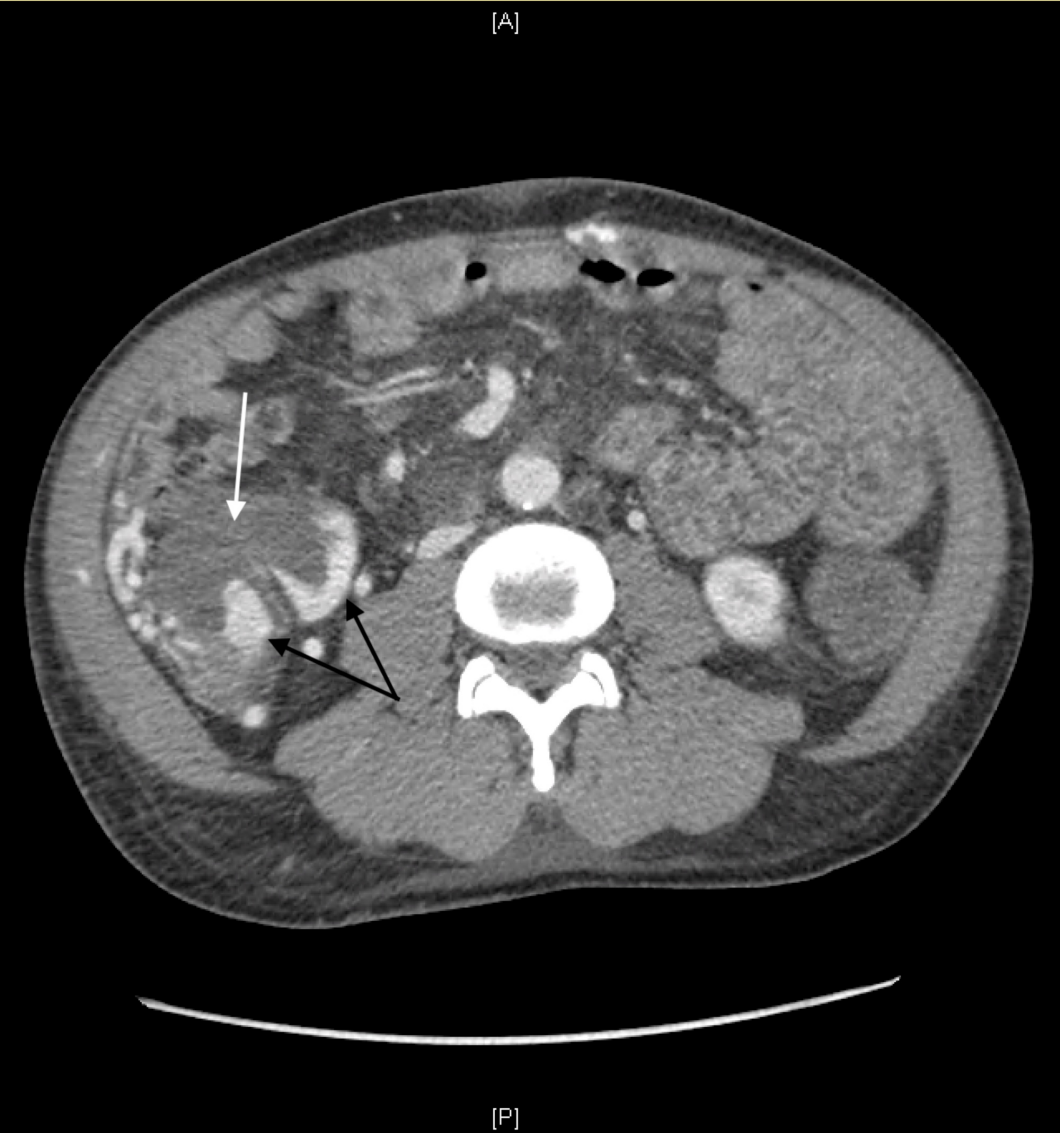
CT abdomen/pelvis with and without IV contrast. Pericecal varices (black arrows) in the right lower abdomen. Extravasation of iodinated contrast into a moderately distended cecum (white arrow) concerning for GI bleed.

**Figure 4:**
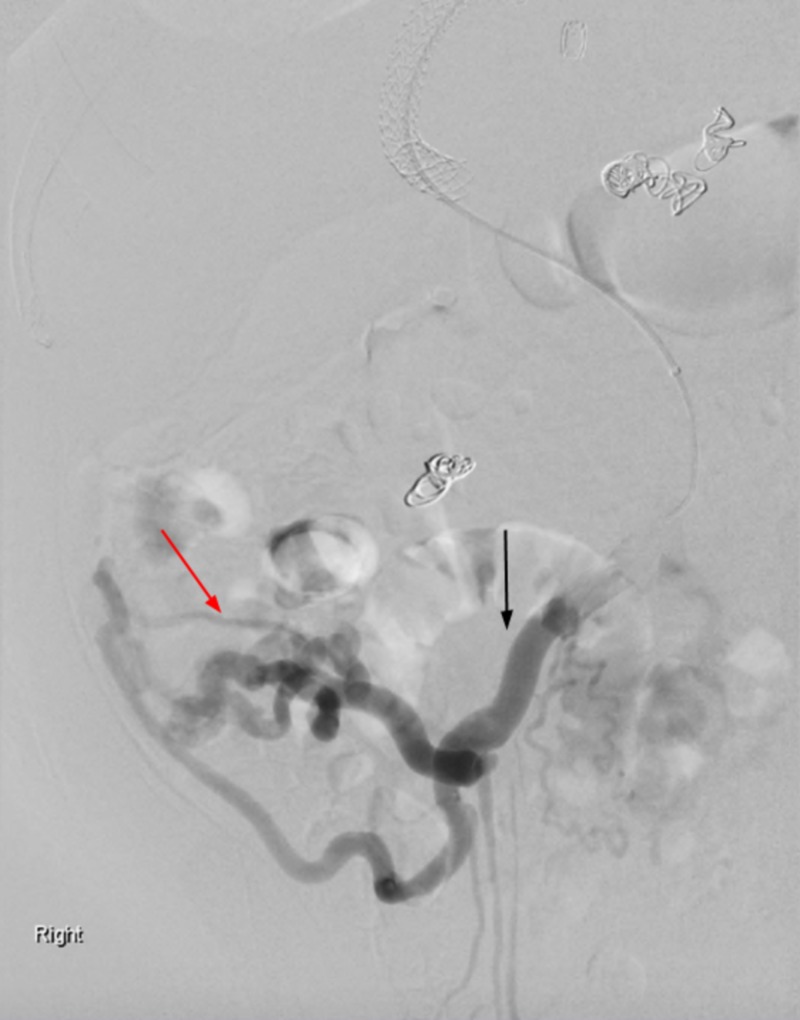
Mesenteric angiogram demonstrating marked enlargement of the superior mesenteric vein (black arrow) with hepatofugal flow. Filling of numerous varicosities in the right lower quadrant with mesocaval shunting through the intercostal veins (red arrow).

**Figure 5:**
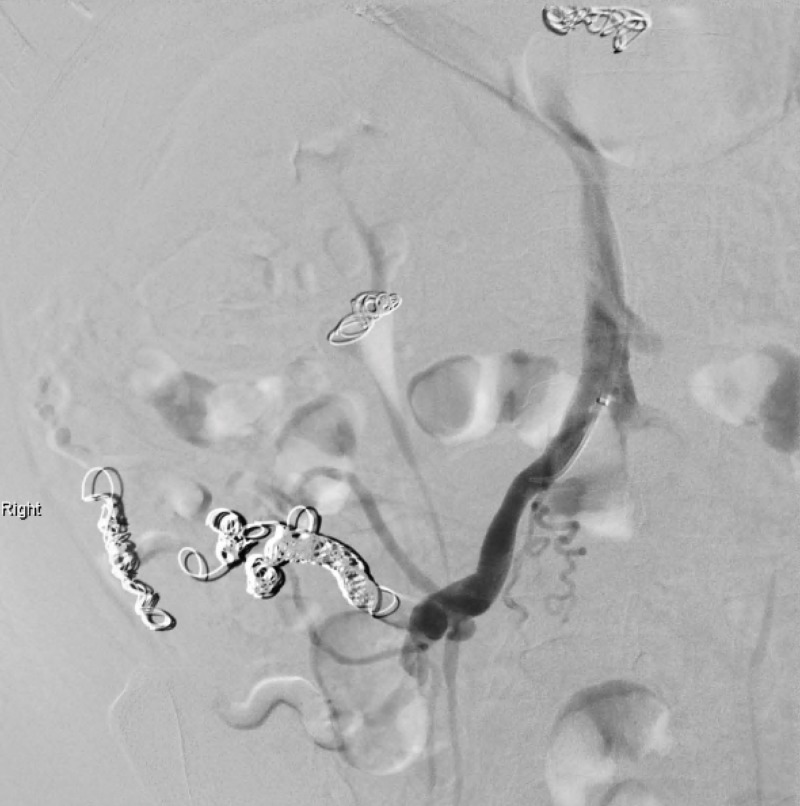
Mesenteric angiogram following embolization of ileocolic and right colic veins.

Given the grave prognosis, the patient decided to transition to palliative care and became no code status for four days. He continued to worsen during this time period, though he later decided he would like to transition off palliative care and after much discussion, he elected to proceed with transjugular intrahepatic portosystemic shunt (TIPS) procedure in an effort to reduce his portal hypertension in hopes of reducing his bleeding risk. Interventional radiology first recommended a triphasic CT scan to better evaluate arterial/venous anatomy relative to cross-sectional anatomy. Triphasic CT scan was performed and demonstrated varicosities throughout the abdomen with a focus of varicosities in the right lower quadrant, likely the right colon (Figure 1). TIPS was performed without complications (Figure 2). Later that evening, the patient developed significant hemorrhage with rectal bleeding; massive transfusion protocol and disseminated intravascular coagulation panel were ordered. Another triphasic CT was performed which demonstrated brisk cecal hemorrhage (Figure 3). The patient was again brought to interventional radiology for an angiogram and embolization of the ileocolic and right colic veins. Mesenteric angiogram demonstrated marked enlargement of the superior mesenteric vein with hepatofugal flow, filling of numerous varicosities in the right lower quadrant, and significant mesocaval shunting (Figure 4). The ileocolic and right colic veins were coil embolized and subsequent venography demonstrated return of hepatopedal flow (Figure 5). Immediately after embolization, the patient’s hemodynamic status improved with normalization of his blood pressure from 80/45 to 115/60. He was transferred back to the ICU in stable condition. Two days later the patient began to develop right lower quadrant pain and his D-Dimer began trending up, which was concerning for possible ischemic colitis; however, this abated after a few hours. He remained an inpatient for an additional five days and on the day of his discharge he was awake, oriented, polite, and cooperative.

During the first nine months of follow-up, the patient has had a complicated course related primarily to his chronic liver disease. He has suffered from intermittent abdominal pain and has been hospitalized or seen in clinic for lactic acidosis, bouts of abdominal pain, an incarcerated right inguinal hernia, significant scrotal edema, and methicillin-resistant Staphylococcus aureus bacteremia. He has, however, attempted positive lifestyle changes, including abstaining from alcohol and illicit drugs and improving his social support. He has had neither recurrent episodes of hematochezia nor has he suffered additional bouts of hepatic encephalopathy. The patient continues to be followed closely as an outpatient.

## Discussion

Ectopic varices are defined as varices outside the cardio-esophageal junction [3]. Approximately five percent of variceal hemorrhages are attributable to ectopic varices [4]. Cecal varices are exceedingly rare and have not been well described in the literature [1]. Moreover, cecal varices are usually found in the context of pan-colonic varices, unlike the case described herein [5]. Cecal varices most often develop secondary to portal hypertension, usually in the context of alcohol-induced liver cirrhosis [2]. While alcohol-induced portal hypertension is the most likely cause of cecal varices, various etiologies of portal hypertension as well as more rare factors may give rise to cecal varices. Pre-hepatic causes of portal hypertension resulting in cecal varices are generally due to portal vein thrombosis [6]. Intrahepatic causes of portal hypertension resulting in cecal varices include viral, toxic, oncologic and metabolic etiologies, with the most common being alcohol induced [2]. Post-hepatic causes of portal hypertension resulting in cecal varices, namely hepatic vein thrombosis, have been identified secondary to myeloproliferative disorders, protein C and/or S deficiency, antithrombin III deficiency, and Factor V Leiden [2]. Other causes of cecal varices include chronic pancreatitis, congestive heart failure as well as unidentified, idiopathic causes [7].

Due to the paucity of data surrounding cecal varices, as well as the various underlying causes, diagnosis and best treatment strategies remain equivocal. Most literature currently points to selective mesenteric angiography as the ideal diagnostic approach as it may allow for precise localization of hemorrhage as well as immediate therapeutic options [2, 8]. However, some authors argue for colonoscopy as the method of choice for diagnosis of cecal varices, at least insofar as to rule out cecal varices with high specificity and possibly treat with band ligation [9-10]. Unfortunately, in the setting of hemorrhage the diagnostic rate of colonoscopy is reduced and patients with bleeding cecal varices are likely to be in a critical condition, making colonoscopy potentially prohibitive [11]. Other treatment options range from resuscitation and observation to total colectomy depending on patient and institutional factors [5]. A recent, multicenter, retrospective study attempted to assess the efficacy of TIPS procedure for patients for ectopic variceal bleeding; however, only one patient from their sample suffered from bleeding cecal varices [3]. That patient did well post-operatively, but there is nonetheless a paucity of data surrounding outcomes in patients with bleeding cecal varices post-TIPS.

Nonbleeding cecal varices have even less available treatment data. One case reported successfully reduced cecal variceal size with the use of propranolol, but the patient could not tolerate the side effects of the medication [5]. Ultimately the underlying cause of the varices must be addressed and all treatments secondary to this goal should be viewed as bridging and stabilization therapies. The curative treatment for cecal varices among patients with portal hypertension due to liver cirrhosis is liver transplant, but due to the relative scarcity of liver transplants the final therapy is usually TIPS procedure [5].

In this case, the decision to embolize the right colic and ileocolic veins post-TIPS procedure was made in an urgent setting to stabilize a hemodynamically unstable patient despite the known risk of potential bowel ischemia. Post-embolization, the patient showed no evidence for bowel ischemia. We postulate that upon embolization of the right and ileocolic veins, the presence of a mesocaval shunt provided venous return from the right colon, which the pressure gradient now favored. Some authors have previously attempted mesocaval shunt procedures as therapy for small intestinal bleeding secondary to portal hypertension [12]. This provides some credence that in patients with bleeding lower gastrointestinal varices secondary to portal hypertension refractory to traditional therapeutic approaches (i.e. TIPS procedure) embolization of the affected venous segments in the presence of mesocaval shunting may be an acceptable therapeutic option.

As demonstrated in this case, colonic varices can be a life-threatening and somewhat illusory cause of gastrointestinal bleeding. The presence of a spontaneous mesocaval shunt may allow safe embolization of the colonic varices when concerned about ischemic bowel. 

## Conclusions

As demonstrated in this case, cecal varices can be a life-threatening and illusory cause of gastrointestinal bleeding. The presence of a spontaneous mesocaval shunt may allow safe embolization of cecal varices when concerned about ischemic bowel.
